# Mycosis Inhibits Cannibalism by *Melanoplus sanguinipes*, *M. differentialis*, *Schistocerca americana*, and *Anabrus simplex*

**DOI:** 10.1673/031.013.12201

**Published:** 2013-11-05

**Authors:** Stefan T. Jaronski

**Affiliations:** USDA Agricultural Research Service, Northern Plains Agricultural Research Laboratory, 1500 N. Central Ave, Sidney, MT 59270

**Keywords:** *Beauveria bassiana*, behavior, biocontrol, grasshoppers, mormon cricket, *Metarhizium acridum*

## Abstract

Cannibalism is common among the Acrididae and the Mormon cricket, *Anabrus simplex* Haldeman (Orthoptera: Tettigoniidae). This behavior has been proposed as a mechanism for the horizontal transmission of Microsporida and entomopathogenic fungi. Aanecdotal observations suggested that the migratory grasshopper, *Melanoplus sanguinipes* Fabricius (Acrididae), and *A. simplex* did not eat cadavers that had been killed by insect pathogenic fungi. The hypothesis tested was that *A. simplex* or *M. sanguinipes* would not cannibalize individuals freshly killed by the entomopathogenic fungi, *Beauveria bassiana* Bals.-Criv. (Vuill.) (Hypocreales: Clavicipitaceae), or *Metarhizium acridum* (Driver and Milner) Bischoff, Rehner, and Humber. Cannibalism was examined in a series of no-choice tests with individual insects. Test insects included healthy adults of *M. sanguinipes*; the differential grasshopper, *M. differentialis* (Thomas); the American grasshopper, *Schistocerca americana* (Drury) (Acrididae); and *A. simplex*. Individual, starved Acrididae or *A. simplex* were confined in small cages with either a fungus-killed (but unsporulated) or uninfected cadaver. The insects were then observed periodically for the first 4 hr. After 24 hr, the cadavers were scored for the degree to which they had been consumed. Very few mycotic cadavers were fed upon by the healthy insects, and, at most only the tarsi were eaten. All four species generally refused to eat fungus-infected cadavers. In contrast, freeze-killed cadavers were partly or entirely consumed by most of the test insects, often within a few hours. Transmission of infection through contact in these tests was between 0–18.9%, depending upon the fungus and insect species, and was lower than the prevalence of cannibalism in all cases.

## Introduction

Cannibalism, defined as “consumption of living or dead members of the same taxon” and more specifically “confamilial feeding of acridids by acridids” (Lockwood 1988), is quite common among the Acrididae and the Mormon cricket, *Anabrus simplex* Haldeman (Orthoptera: Tettigonidae) (Lockwood 1988, 1989; O'Neill et al. 1993, 1994; Simpson et al. 2006). This behavior has been proposed as a mechanism for the horizontal transmission of Microsporida (Streett and Woods 1993) and the secondary transmission of the entomopathogenic fungus *Metarhizium acridum* (Driver & Milner) Bischoff, Rehner & Humber (Hypocreales: Clavicipitaceae) among locusts (Thomas et al. 1995).

The fungi *M. acridum* and *Beauveria bassiana* (Balsamo-Criv.) Vuillemin have been developed for the microbial control of acridids, the former in the USA and the latter in Africa and Australia (Lomer et al. 2001). Both are known to produce conidia both inside (Arthurs et al. 2003) and on the exterior of acridid cadavers. The former process does not require as prolonged and high humidities as the latter. Thus, cannibalism/necrophagy can be a potential route for transmission of these pathogens in the absence of environmental conditions favorable for external sporulation.

After anecdotal observations that the migratory grasshopper, *Melanoplus sanguinipes* (Fabricius) (Orthoptera: Acrididae), and *A. simplex* did not eat cadavers that had been killed by *B. bassiana* during bioassays of entomopathogenic fungi, the hypothesis examined was whether or not *B. bassiana*- or *M. acridum*-killed (but not yet sporulating) insect cadavers would be consumed by healthy individuals, and if contact with such cadavers could transmit fungal infection.

## Materials and Methods

Four species of Orthoptera were observed for cannibalism: adult, nondiapause *M. sanguinipes* from the USDA ARS colony in Sidney, MT, USA; the differential grasshopper, *M. differentialis* Thomas (Acrididae), collected in Fairview, MT, as 5^th^ and 6^th^ instars and reared to adulthood; adult American grasshoppers, *Schistocerca americana,* Drury from a USDA ARS colony; and *A. simplex* collected near Lodge Grass, MT, as 5^th^ and 6th instars and reared to adulthood. All insects were tested as 1–2 week old adults. Two entomopathogenic Ascomycetes were used: *B. bassiana* Strain GHA (Laverlam International, www.laverlamintl.com) and *M. acridum* Strain FI985 (Becker Underwood, www.beckerunderwood.com). Both were obtained as dry conidia powders from the respective manufacturers. The *B. bassiana* strain was registered for use against grasshoppers on rangeland and improved pasture, while the *M. acridum* FI985 was registered in Australia for the control of locusts and grasshoppers and is under development for that use in several Asian countries.

Infected cadavers were created by topical application of an LD_90_–LD_99_ dose for each fungus, the respective dose being based on previous bioassays. Dosing was performed by applying a 1 µL droplet of fungus conidia suspended in vegetable oil to the arthrodial area of the right foreleg coxa. The insects were then incubated in groups at 27° C under a 16:8 L:D photoperiod until death (generally within 5–6 days). Cadavers were collected daily, placed in sealed tubes to prevent cadaver desiccation, and refrigerated at 3° C. Cadavers were used within 48 hr of death. No external signs of mycosis beyond color change (a pink color with *B. bassiana* and a more intense, red color with *M. acridum*) were permitted. Healthy cadavers were created by freezing unexposed insects, thawing them, incubating them at room temperature for 12 hr, and then refrigerating them for 2 days before use, to parallel processing of infected cadavers and to control for a potential confounding effect of freezing. In an ancillary experiment, 12 each of *Beauveria*-infected *M. sanguinipes* and *M. differentialis* adults were frozen and processed in the same manner as the healthy insects and then offered to healthy grasshoppers. In all cases, the dead insect was the same species as the live, test insect.

**Figure 1.**
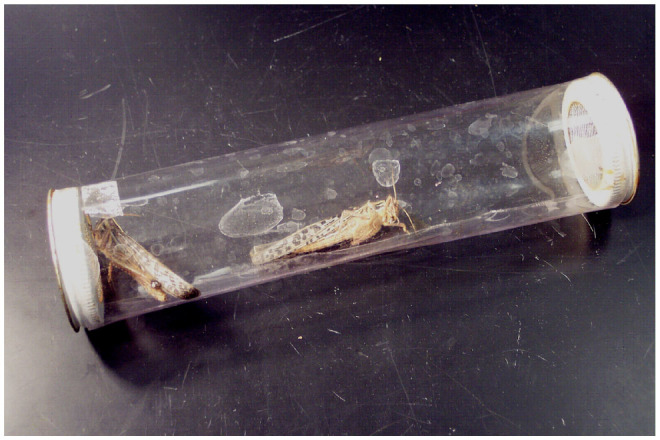
Arena used to test repellency of cadavers. Pictured is an adult *Schistocerca americana* (left) confined with a conspecific cadaver (center). High quality figures are available online.

**Figure 2.**
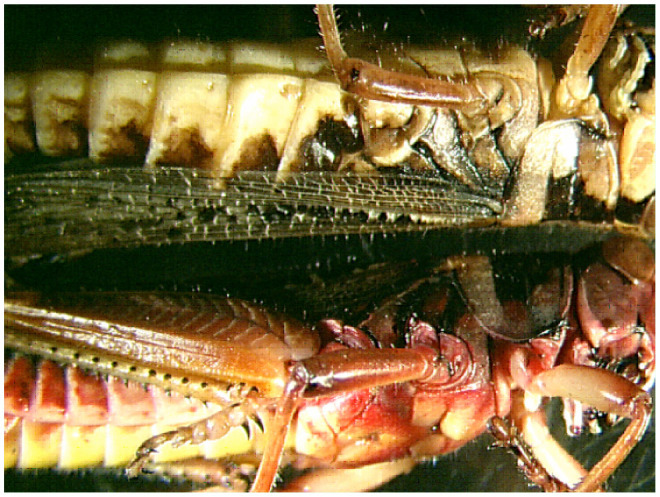
Appearance of cadavers used in the study: (top) healthy cadaver; (bottom) cadaver resulting from *Metarhizium acridum* infection. High quality figures are available online.

Individual healthy grasshoppers were isolated and starved overnight before use. For cannibalism tests, each healthy or fungus-infected cadaver was placed in the center of a 20-cm long, 5-cm diameter, cellulose acetate cylinder with mesh-capped ends. Each live test insect was anesthetized by exposing it to 5–7° C temperatures for 30 minutes, after which it was placed at one end of a tube. The tubes were subsequently arranged in alternating treatments directly beneath incandescent lights. The air temperature during the exposure period was 26–28° C. Equal ratios of male and female test insects were used throughout. Cadavers were conspecific with the live insects tested.

The living insects were periodically observed for their behavior (feeding on cadaver, location in the arena, etc.) during the first 4 hr of exposure to cadavers. Cadavers in the tubes were visually examined and scored for cannibalism 24 hr after introduction into the tubes. Cannibalism was scored as: (0) no visible signs of feeding; (+/-) very slight feeding, which was usually confined to the tarsi of the dead insect, sometimes the antennae; and (+) considerable feeding, which ranged from consumption of part of the abdomen to almost entire consumption of the cadaver. After 24 hr, the cadavers were removed, checked for desiccation, and subsequently incubated at 95–100% RH and 28° C for 4–5 days to verify presence of mycosis (emergence of the fungus from within the cadaver, usually through less sclerotized cuticle and orifices, and eventual covering of the cadaver with mycelium and conidiation characteristic of each fungus). The check for desiccation was intended to identify a potential bias in attractiveness of uninfected or infected cadavers.

After the contact period, the live test insects were placed in clean cellulose acetate tubes and incubated at 27–28° C for 21 days. They were fed daily with lettuce ad libitum. Mortality was recorded daily. Any insects found dead were decontaminated by a 1 min immersion in 0.5% NaOCl, followed by two rinses in deionized water, and subsequently incubated at 95–100% RH to elicit any mycosis present (Lacey and Brooks 1997).

Each test used 10–24 insects per treatment, depending upon availability, and was replicated in entirety twice.

### Statistical Analysis

The relative proportions of the feeding categories described above were subjected to multinomial tests using Statistix 9 (Analytical Software, Inc, www.statistix.com).

## Results

All four species readily consumed healthy (uninfected) cadavers, often within 4 hours. Typically the abdomen, head, and/or thorax were largely consumed after 24 hr. Cannibalism of infected cadavers was minimal even after 24 hr. Generally, only the tips of tarsi were eaten. The observations are presented in Table 1. None of the cadavers had dried out during the exposure period (and thus presumably did not lose attractiveness or repellency), and all displayed mycosis by the corresponding fungus within. No uninfected cadavers had outgrowth by either fungus. Comparison of feeding upon frozen then thawed *Beauveria*infected *M. sanguinipes* and *M. differentialis* cadavers with those that were normally processed revealed no significant differences between the two processing methods (χ^2^ = 0.38, d.f. = 1, *p* = 0.54 for *M. sanguinipes*, χ^2^ = 0.25, d.f. = 1, *p* = 0.62 for *M. differentialis*). There was no normal feeding observed, just the partial feeding seen in the main experiment, indicating that there was no effect from the freeze-thaw processing of healthy grasshoppers.

**Table 1.**
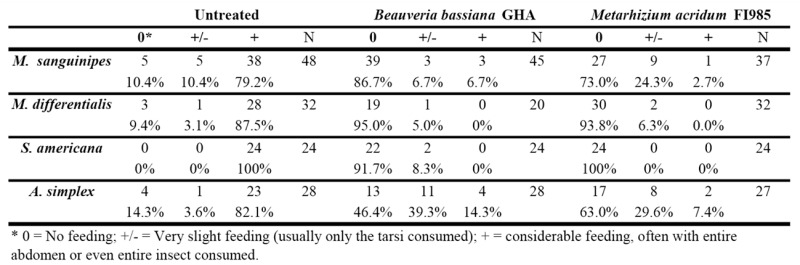
Influence of the presence of internal mycosis on the cannibalism by three Acrididae and one Tettigonidae.

During the first 4 hr of the observation period, there was generally little activity by the live insects. About half of each species remained at or near their original positions after they had recovered from their cold anesthesia. A few of each species proceeded to the uninfected cadaver and probed it with their antennae, mandibles, and maxillae; none did so when the cadaver had been killed by fungus infection. Only a total of six of the Mormon crickcrickets in both replicates began to feed on the abdomens of uninfected cadavers.

There were no significant differences among replicate tests and between males and females in each test, so all data for each species were combined for analysis. In all cases, cannibalism of fungus-infected cadavers was significantly less than cannibalism of healthy cadavers (multinomial test, *p* < 0.001). Data for *S. americana* could not be analyzed be cause of the absolute differences in feeding (Table 1). In the case of *M. sanguinipes*, there was a significant difference in the degree of cannibalism between the two fungi (χ^2^ = 50.68, d.f. = 2, *p* < 0.001), with a greater proportion of *Beauveria*-infected cadavers fed upon than *Metarhizium*-infected bodies, especially in terms of slight feeding. A similar sitsituation existed with *A. simplex* (χ^2^ = 12.48, df = 2, *p* = 0.002), with greater cannibalism on *Beauveria*-infected cadavers, but not with *M. differentialis* or *S. americana*. These last two species did not show significant differences in aversion to the two fungi.

**Table 2.**
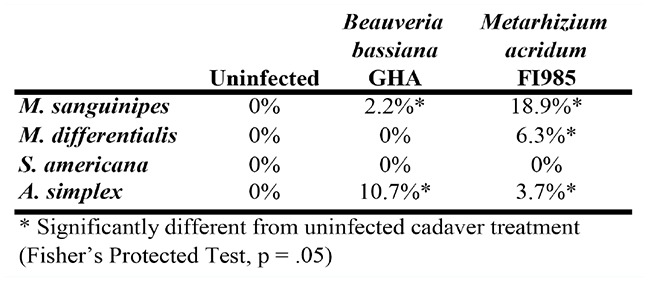
Fungus infection rates among insects exposed to fungus-killed cadavers in no choice tests.

Fungal transmission occurred only among insects exposed to fungus-killed cadavers, but was very low (Table 2). Light feeding did not transmit *B. bassiana* strain GHA readily. Prevalence of mycosis after 21 days was 0–10.7% among the four species dying after feeding on *Beauveria*-infected cadavers. Transmission of *M. acridum* FI985 was higher, 0–18.8%, depending on the species. There were no infections among insects that did not feed on an infected cadaver.

## Discussion

Feeding on healthy cadavers was considerable and occurred within a few hours regardless of the species or sex of the cadaver or live insect (Table 1). This result is in marked contrast to observations of Bomar and Lockwood (1994), where *M. sanguinipes* responded indifferently to volatiles from cadavers, and starved female *M. differentialis* differences could be attributed to use of a Y-tube olfactometer choice tests with air as a control. In my experiments, the insect was confined with the cadaver, allowing exploration of the cadaver by antennal contact and palpation, with an end result of cannibalism or not. The Bomar and Lockwood (1994) experiments were solely concerned with long (70 cm) olfactory response to a cadaver.

Cannibalism in all four species was significantly inhibited by mycosis. If the cadaver was infected by either fungus, feeding was greatly delayed and considerably reduced in extent. In many cases, the live insect approached the infected cadaver and explored it with its antennae for some moments, but then backed away and did not feed, even though it had been starved for the previous 24 hr and was closely confined with the cadaver. In a number of cases, especially with *A. simplex* but also with *M. sanguinipes* with *M. acridum* FI985, the insects ate only the tarsi or antennae of the cadaver, leaving the rest untouched. Of note is that repellency was not absolute, even when the insects had been previously starved. A small proportion of *M. sanguinipes* (2.7–6.7%) and *A. simplex* (7.4–14.3%) did feed on fungus-infected cadavers, consuming some part of the cadavers' abdomen or head. The cadavers in question developed external mycoses of the respective fungi. This aversive behavior indicates that the four species could detect the presence of the mycosis and therefore avoided or minimized contact with an infected cadaver.

Some infection did occur subsequent to feeding upon the cadaver. It should be noted that neither fungus had sporulated to any great degree within the cadavers, based upon visual examination of the cadavers after the exposure period. The fungi remained as partially moist mycelium within the cadaver body cavities. It is not clear how widespread this aversive behavior is within the Acrididae. Both *M. sanguinipes* and *M. differentialis* are within the subfamily Melanoplinae, which is noted for species with polyphagy. *Schistocerca americana* is within the subfamily Cyrtacanthacridinae, also noted for polyphagous and cannibalistic species. *A. simplex*, a tettigonid, is known for its cannibalistic and general necrophagic behavior (Simpson et al. 2006). Cannibalism by Acrididae from other subfamilies has been observed in the field (Lockwood 1988, 1989), although in olfactometer experiments *Camnula pellucida* and *Hadrotettix trifasciatus* (Oedipodinae), *Aulocara elliotti* (Gomphocerinae), and *Brachystola magna* (Romaleidae), had generally indifferent and variable necrophiliac responses (Bomar and Lockwood (1994).

The repellant character of entomopathogenic fungi is known for several insects. Dead, sporulated *Blatella germanica* cadavers were not cannibalized, unlike uninfected cadavers, and were far less efficient transmission vectors (Kaakeh et al 1996). Perhaps the most notable example is the repellency of *Metarhizium* conidia for grubs of *Popillia japonica* in soil (Villani et al. 1994). In a subsequent study, Fry et al. (1997) observed that Japanese beetle grubs avoided soil containing *M. anisopliae* mycelial particles beginning 48-96 hr after incorporation, presumably as the mycelium had rehydrated and begun metabolizing. Tawny mole crickets, *Scapteriscus vicinus*, were repelled by both *B. bassiana and M. anisopliae* conidia in soil (Villani et al. 2002). Repellency of *M. anisopliae* and *B. bassiana* has also been observed with some termites, namely *Coptotermes lacteus* (Staples and Milner 2000) and *Macrotermes michaelseni* (Mburu et al. 2009). In the latter study, the termites were able to detect and avoid the presence of fungus conidia from a distance, and their aversive behavior was directly proportional to the concentration of conidia in the soil. These data implicate release of volatile organic compounds from the conidia and diffusion through the soil air spaces, compounds that were detectable by termite olfaction. There seems to be considerable variation in the degree of repellency among strains of *M. anisopliae* (Staples and Milner 2000). Mburu et al. (2009) claimed an inverse relationship between fungal virulence and repellency, in addition to demonstrating clear differences between *M. anisopliae* and *B. bassiana* in repellency, at least among the isolates they tested.

Acrididae are clearly olfactory-driven insects, using volatile organic compounds to locate food plants (Hopkins and Young 1990; Kang et al. 1995) and dead insects (Bomar and Lockwood 1994). The latter authors demonstrated that seven grasshopper species were necrophiliac to a varying degree, while for Lockwood (1988) the behavior of grasshoppers in the vicinity of cadavers clearly indicated an olfactory response. Thus, grasshoppers clearly possess the physiological ability to detect volatile organic compounds emanating from fungus-killed cadavers.

Entomopathogenic fungi are known to produce a range of volatile organic compounds. *Beauveria bassiana* was observed to produce diisopropyl naphthalenes, ethanol, and sesquiterpenes (Crespo et al. 2008). Hussain et al. (2010) observed that *B. bassiana* and *M. anisopliae* produced a wide variety of C7–C19 hydrocarbons, and that the nature of the volatile organic compound mixture differed not only between fungus species (and between the two *M. anisopliae* isolates) but also between *in vitro*- and *in vivo*-derived cultures within the same isolate. The responsible volatiles involved in Acridid repulsion remain to be identified, but with the data of Crespo et al. (2008) and Hussain et al. (2010) there is a starting point of volatile organic compound evaluation using electroantennogram methods, e.g., Chen and Kang (2000).

The aversive behavior of grasshoppers to mycotic cadavers can minimize the recycling potential of entomopathogenic Ascomycetes in operational use. While this situation may be perceived as a disadvantage in implementing these pathogens for grasshopper control, it is an advantage from the perspective of regulators concerned about the importation of nonindigenous *M. acridum* into North America and ecologists concerned with broad, persistent effects of an entomopathogenic fungus on grasshopper populations (Lockwood 1993a, 1993b; Branson et al. 2006). External conidiation on grasshopper cadavers in the field is very rare, especially under North American plains conditions (S. Jaronski, personal observation), because the process requires continuous high humidity for several days. Internal conidiation can be a possible transmission mechanism, but the averse behavior described here, as well as the rapid removal of dead grasshoppers by ubiquitous ants and other scavengers (S. Jaronski, personal observation; Arthurs et al. 2001), should minimize this potential.
